# Demineralized Freeze-Dried Bovine Cortical Bone: Its Potential for Guided Bone Regeneration Membrane

**DOI:** 10.1155/2017/5149675

**Published:** 2017-08-29

**Authors:** David B. Kamadjaja, Achmad Harijadi, Pratiwi Soesilawati, Eny Wahyuni, Nurul Maulidah, Akhsanal Fauzi, Fika Rah Ayu, Roberto Simanjuntak, R. Soesanto, Djodi Asmara, Andra Rizqiawan, Peter Agus, Coen Pramono

**Affiliations:** ^1^Department of Oral and Maxillofacial Surgery, Faculty of Dental Medicine, Universitas Airlangga, Surabaya, Indonesia; ^2^Department of Oral Biology, Faculty of Dental Medicine, Universitas Airlangga, Surabaya, Indonesia; ^3^Residency Program, Oral and Maxillofacial Surgery, Faculty of Dental Medicine, Universitas Airlangga, Surabaya, Indonesia

## Abstract

**Background:**

Bovine pericardium collagen membrane (BPCM) had been widely used in guided bone regeneration (GBR) whose manufacturing process usually required chemical cross-linking to prolong its biodegradation. However, cross-linking of collagen fibrils was associated with poorer tissue integration and delayed vascular invasion.

**Objective:**

This study evaluated the potential of bovine cortical bone collagen membrane for GBR by evaluating its antigenicity potential, cytotoxicity, immune and tissue response, and biodegradation behaviors.

**Material and Methods:**

Antigenicity potential of demineralized freeze-dried bovine cortical bone membrane (DFDBCBM) was done with histology-based anticellularity evaluation, while cytotoxicity was analyzed using MTT Assay. Evaluation of immune response, tissue response, and biodegradation was done by randomly implanting DFDBCBM and BPCM in rat's subcutaneous dorsum. Samples were collected at 2, 5, and 7 days and 7, 14, 21, and 28 days for biocompatibility and tissue response-biodegradation study, respectively.

**Result:**

DFDBCBM, histologically, showed no retained cells; however, it showed some level of in vitro cytotoxicity. In vivo study exhibited increased immune response to DFDBCBM in early healing phase; however, normal tissue response and degradation rate were observed up to 4 weeks after DFDBCBM implantation.

**Conclusion:**

Demineralized freeze-dried bovine cortical bone membrane showed potential for clinical application; however, it needs to be optimized in its biocompatibility to fulfill all requirements for GBR membrane.

## 1. Introduction

Reconstruction of alveolar bone defect required bone grafting procedure [1, 2]; however, to improve the bone regeneration it was important to keep the grafted defect separated from fibrous organization by inserting membranes following the principle of guided bone regeneration [3, 4]. Collagen from bovine pericardium had been widely used as resorbable membranes material because of its biocompatibility, hemostatic activity, and tissue integration [5]. As a type of native collagen, bovine pericardium collagen could be rapidly resorbed; therefore its manufacturing process usually involved chemical cross-linking to prolong its biodegradation. However cross-linking process of the collagen fibrils was associated with poorer tissue integration and delayed vascular invasion. In addition, an increased invasion of inflammatory cells had been observed after implantation of chemically cross-linked collagen [6].

In view of this, it was necessary to obtain an alternative type of membrane which had features that was comparable to and could overcome the disadvantages of pericardium membrane. This study attempted to explore the potential of demineralized freeze-dried bovine cortical bone (DFDBCB) to be used as a guided bone regeneration membrane. As this membrane was expected to be used as xenogeneic biomaterial in humans, it was important to determine that it was biocompatible, which meant that it should not cause antigenicity, cytotoxicity, and excessive immune response. Besides, in order to be clinically effective as a barrier membrane it should not cause abnormal tissue response or undergo too early degradation. This study was aimed to analyze cytotoxicity, antigenicity, immune and tissue response, and biodegradation behavior of DFDBCB membrane.

## 2. Materials and Methods

### 2.1. DFDBCB Membrane Manufacturing Process

DFDBCBM processing was performed at Tissue Bank/Center for Biomaterial and Stem Cell, Dr. Soetomo General Hospital, Surabaya, as follows. Bovine cortical bone was immersed in 3% hydrogen peroxide solution to remove blood, fat, and bone marrow. The solution was replaced daily until the bone turned white and no trace of fat and marrow was detected after which the bone was washed out by soaking in daily replaced, sterile distilled water for 5 to 6 days. The cortical bone was then cut up into pieces with band saw under sterile condition. Demineralization was performed by immersing the bone in 0.1% HCL solution until the desired flexibility of the bone was achieved. The excess of HCL was subsequently washed out by soaking the “soft bone” in sterile distilled water many times until neutral pH was achieved, checked with pH meter. The demineralized bone was then cut into layers of membrane with 300 *μ*m thickness using special microtome. Freeze drying was done by freezing for at least 24 hours and subsequently dried for 18–24 hours until less than 5% water content was achieved, followed by double packaging and sterilization using gamma irradiation.

### 2.2. In Vitro Anticellularity Evaluation

Immunogenic potential evaluation was to evaluate the decellularization of DFDBCB membrane compared to the widely used bovine pericardium membrane or BPCM in brief (Jason Membrane®, Botiss, Germany). Sixteen samples of 5 × 5 mm DFDBCBM and BPCM were fixed in 10% buffered formaldehyde for 3 days and subsequently embedded in paraffin. Sections, 5 *μ*m thick, were deparaffinized with xylene, rehydrated in 100% alcohol, and washed in distilled water and then stained with Hematoxylin-Eosin. The histology analysis for any retained cells (osteoblasts and osteocytes) was performed with 400x magnification.

### 2.3. In Vitro Cytotoxicity Test

Cytotoxicity test was performed by exposing human gingival fibroblast culture to DFDBCBM-conditioned medium and analyzed with MTT Assay as follows. Human gingival fibroblast (Stem Cell Laboratory, Institute of Tropical Disease, Universitas Airlangga) was grown in normal medium (containing *α*-MEM, FBS, antibiotic, and antifungal) in 96-well microplate at cellular density of 3 × 10^3^ cell/well. Conditioned-medium was made by rehydrating DFDBCB membrane in normal medium mentioned above at a concentration of 1 gram/mL and incubated at 37°C. The membranes were subsequently removed after 24, 48, and 72 hours and the medium was ready for use. The normal medium in 5 wells of experimental group was removed and replaced with DFDBCBM-conditioned medium, while in the remaining 5 wells the normal medium was retained as control group. The wells were then incubated at 37°C for 20 hours. For cytotoxicity test medium in all wells was replaced with MTT reagent as much as 5 mg/mL for 4 hours, to which DMSO, 200 *μ*L/well, was subsequently added. The optical density of the dye absorption was analyzed with Elisa reader at the wavelength of 595 nm. Cytotoxicity was determined by percentage of viable cell being under 60%.

### 2.4. Surgical Procedure

Surgical procedure for this study was done at operating room at Laboratory of Biochemistry, Faculty of Medicine, Universitas Airlangga, Surabaya, which had been approved by Commission on Ethical Clearance, Faculty of Dental Medicine, Universitas Airlangga, Surabaya.

### 2.5. In Vivo Immune Response Evaluation

**Figure 1:**
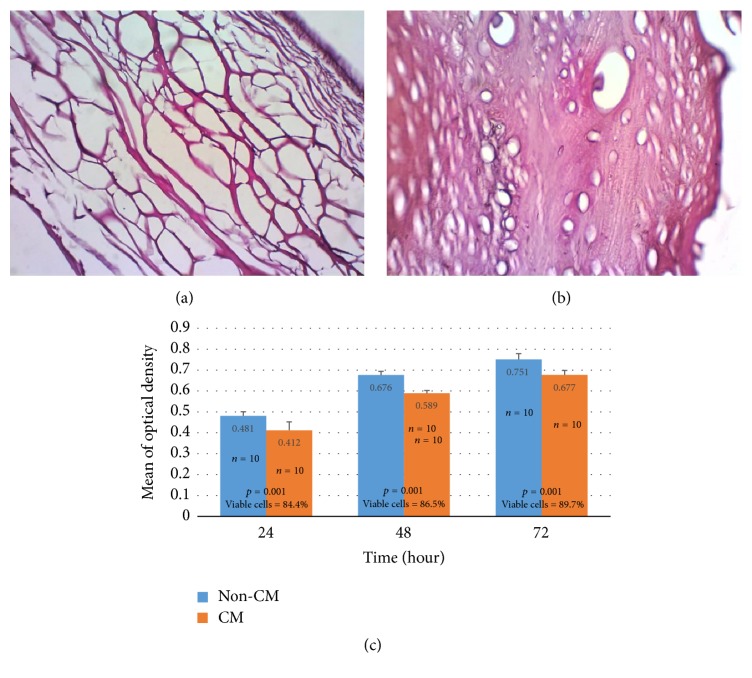
Micrograph of BPCM (a) and DFDBCBM (b). Collagen fibers of BPCM were seen to form loose structures giving impression of membrane porosities. On the other hand, DFDBCBM demonstrated dense collagen fibers with lacunas which were characteristic features of cortical bone. No retained cell was found in either type of membrane (Hematoxylin-Eosin, ×400 magnification). The result of MTT Assay (c). The percentage of viable cells after exposure of human gingival fibroblast culture to DFDBCBM-conditioned medium was 84.4%, 86.5%, and 89.7% after 24, 48, and 72 hours of exposure, respectively (CM = conditioned medium; non-CM = non conditioned medium).

Thirty male Wistar rats used in this study were randomly divided into 2 groups. In experimental group 5 × 5 mm DFDBCBM were implanted in the rats' dorsal subcutaneous tissue while in control group BPCM (Jason Membrane®, Botiss, Germany) was used for the implantation (Figure 1). Five rats from each group were sacrificed at 2, 5, and 7 days after implantation for histology examination. The number of polymorphonuclear or PMN cells, macrophages, and lymphocytes adjacent to implanted membranes was counted to quantitatively determine the immune response to DFDBCBM and BPCM subcutaneous implantation. The inflammatory cells counting was performed “blind”; that is, the microscopic areas chosen were randomly assigned, done by two different persons, and the slides numbers were randomized to make the counting blinded.

### 2.6. Tissue Response and Biodegradation Evaluation

Forty male Wistar rats used in this study were randomly divided into 2 groups. A 5 × 5 mm BPCM (Jason Membrane®, Botiss, Germany) and DFDBCBM were subcutaneously implanted in rat's dorsum as control and experimental group, respectively. Five samples from each group were sacrificed at 7, 14, 21, and 28 days after implantation for histology examination followed by histomorphometry analysis to evaluate tissue response and biodegradation behavior of the membranes.

### 2.7. Histological Examination Methods

Experimental animals were sacrificed at the end of implantation period by over sedating them with ether vapor. The implanted membranes were retrieved by removing the membrane together with their surrounding tissues, fixed in 10% buffered formalin solution. The tissue was then embedded in paraffin block and thin section was made using microtome. The sections were then stained with Haematoxylin & Eosin and investigated with light microscope. Microscopic sections were evaluated for histologic evaluation and histomorphometry analysis.

Histomorphometry analysis was performed using grading scale developed by Jansen et al. [7] in which the capsules formed around the implanted membrane were evaluated using semiquantitative and semiqualitative grading scales (Table 1). Semiquantitative classification consisted of capsule thickness quantification based on the number of observed fibroblast. The semiqualitative rating evaluated the capsule and the interface tissue consisting of numerical rating of the tissue morphology (fibrous tissue, fat tissue, and maturity) and cellularity (presence of fibroblasts, macrophages, giant cells, and other inflammatory cells), respectively. The quantification of the degradation rate was performed based on the mean thickness of the residual membranes measured at five interval points as developed by Moses et al. [8].

### 2.8. Statistical Analysis

Statistical analysis was performed using software package IBM SPSS for Windows version 21. The data collected from in vivo immune response evaluation was analyzed using one-way analysis of variance. The data collected from semiquantitative and semiqualitative evaluation of the capsules were analyzed with Kruskal–Wallis. Statistical significance was determined when the *p* value < 0.05.

## 3. Result

### 3.1. Result of Anticellularity Evaluation

The result of in vitro anticellularity evaluation showed that no retained cell was found in H&E staining of all samples of DFDBCBM (Figure 1). The result confirmed that the manufacturing process of DFDBCBM had removed the key cellular components of cortical bone, that is, osteoblasts and osteocytes. This would mean that DFDBCBM, to a certain level, had no antigenicity potential to recipient tissue after xenogeneic implantation.

### 3.2. Result of Cytotoxicity Test

The result of MTT Assay indicated that there was statistical difference (*p* < 0.001) in optical density between fibroblast exposed to conditioned-medium and those in normal medium after 24, 48, and 72 hours of incubation. The result also showed that percentage of viable fibroblast was 84.44%, 86.46%, and 89.67% upon exposure to DFDBCBM-conditioned medium for 24, 48, and 72 hours, respectively (Figure 1).

### 3.3. Result of In Vivo Immune Response Evaluation

**Figure 2:**
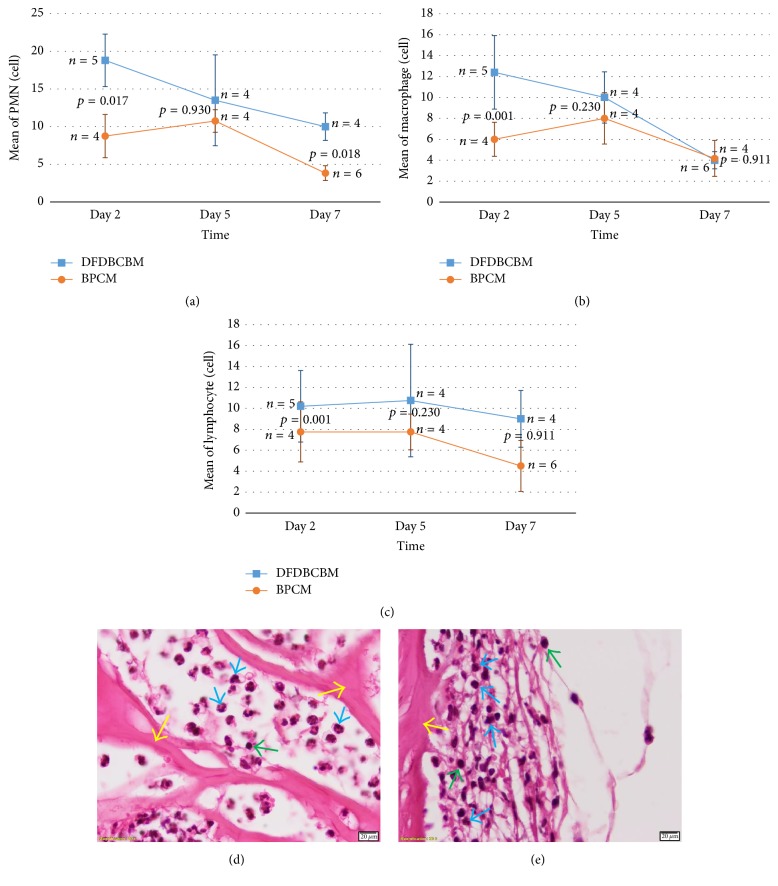
Distribution of inflammatory cells infiltration following subcutaneous implantation of BPCM and DFDBCBM at 2, 5, and 7 days after implantation. Mean of PMN count (a) in DFDBCBM group was significantly higher than BPCM at days 2 and 7, whereas mean of macrophage (b) and lymphocyte (c) count in DFDBCBM group was significantly higher than BPCM at day 2 after implantation. All inflammatory cells infiltration exhibited downward pattern from day 5 to day 7 indicating no prolonged inflammation. Microscopic picture of inflammatory cell infiltration at day 2 following subcutaneous implantation of BPCM (d) and DFDBCBM (e). PMN and lymphocyte were the predominant inflammatory cells seen in the tissue surrounding both membranes; blue arrow head pointing to PMN, green to lymphocytes, and yellow to respective membrane structure (H&E staining, ×1,000 magnification).

Histology examination showed that the characteristic of immune response was somewhat different between the two groups. Infiltration of inflammatory cells was evident at the periphery of DFDBCBM while in BPCM inflammatory cells were found both in the periphery and inside the membrane porosities. Intramembrane cell infiltration was more evident in later days after implantation, whereas in DFDBCBM cleavage of membrane structure was noted at day 7 after implantation (Figure 2).

The result of histology cell counting showed that the amount of PMN cells in DFDBCBM group was significantly higher than BPCM group on days 2 and 7 (*p* < 0.05) except for day 5 (*p* > 0.05). The histogram exhibited that the amount of PMN showed downward or declining pattern in both groups along the time of examination (Figure 2).

The result demonstrated that macrophage counting was significantly higher in DFDBCBM group than BPCM group (*p* < 0.05) at day 2; however, no statistical difference was found in the amount of macrophage between the two groups (*p* > 0.05) at days 5 and 7 after implantation. The histogram showed that the amount of macrophage in DFDBCBM group showed consistent downward trend as opposed to BPCM group which showed fluctuating pattern (Figure 2).

The result also revealed that lymphocyte counting was significantly higher in DFDBCBM compared to BPCM group (*p* < 0.05) at day 2 after implantation; however, no statistical difference was found in the amount of lymphocyte between the two groups (*p* > 0.05) at days 5 and 7 after implantation. The histogram showed that the amount of lymphocyte showed downward or declining pattern in both groups along the time of examination (Figure 2).

### 3.4. Result of Tissue Response Evaluation

**Figure 3:**
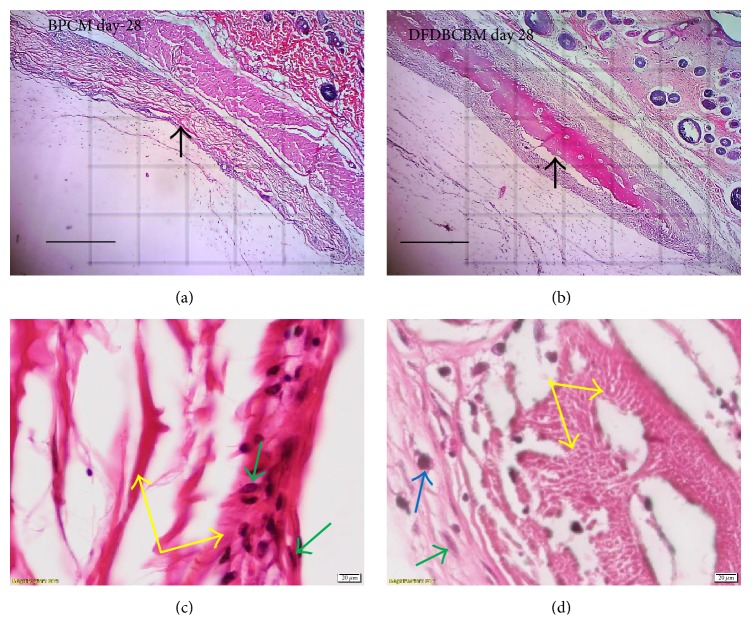
Microscopy of tissue response following subcutaneous implantation of BPCM and DFDBCBM. Both membranes were surrounded by fibrous capsule of 10–20 cell thickness. There was no sign of BPCM (a) degradation whereas some cleavages were noted in DFDBCBM (b) indicating degradation at day 28 after implantation (arrow pointing to membrane and their surrounding capsule, H&E staining, ×40 original magnification, bar = 50 *μ*m). The interface tissues surrounding BPCM (c) and DFDBCBM (d) exhibiting fibrous tissue consisting fibroblasts with scattered foci of macrophages (green arrow pointing to fibroblast, blue to macrophage, and yellow to the respective membrane, H&E staining, ×1,000 magnification).

Examination of the histological sections revealed a characteristic and somewhat uniform tissue response without signs of prolonged inflammatory reaction in BPCM and DFDBCBM groups. Fibrous capsule surrounding the membranes was from few cells thick in the initial phase of healing (day 7) to approximately 20–30 cells thick in later healing stage (Figure 3). The capsule contained more fibroblasts as primary cellular component in early phase but turned to be more fibrotic, with few fibrocytes, in later stage indicating maturity of the capsules. The capsules, in some area of the membranes, made direct contact with the membrane surface without the presence of layers of reactive cells but in majority there existed layers of fibrous capsules containing macrophages and foreign body giant cells which in this study was referred to as interface tissue (Figure 3).

**Figure 4:**
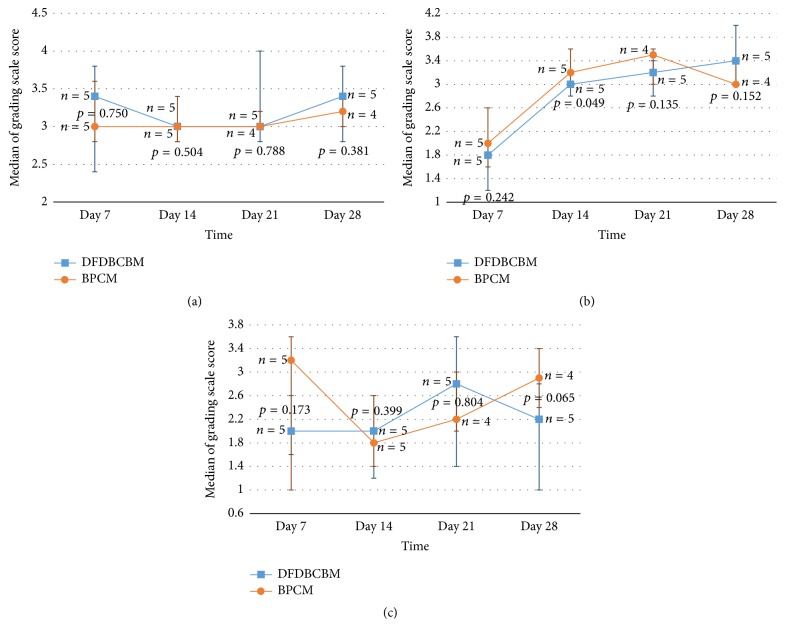
Analysis of tissue response following subcutaneous implantation of BPCM and DFDBCBM. There was no significant difference in capsule quantity (a), capsule quality (b), and interface quality (c) between the two groups throughout the observed healing periods.

The data of fibrous layer quantification showed that the median score of DFDBCBM group was higher than that of BPCM group in early phase (day 7) and late phase (day 28) (Figure 4); however there was no statistical difference in the observed variable (*p* > 0.05) between the two groups.

The data of fibrous layer quality showed that the median score of both BPCM and DFDBCBM group showed upward trend from early phase towards intermediate and late phase of healing (Figure 4). Statistical analysis showed that that there was no difference in the observed variable (*p* > 0.05) between the two groups.

The data of fibrous layer quality showed that the median score of both BPCM and DFDBCBM group showed upward trend from early phase towards intermediate and late phase of healing (Figure 4). Statistical analysis showed that there was no difference in the observed variable (*p* > 0.05) between the two groups.

### 3.5. Result of Biodegradation Evaluation

**Figure 5:**
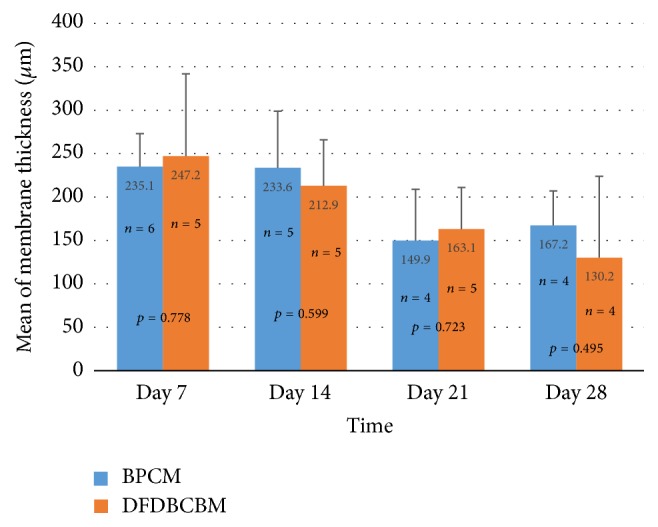
Measurement of membrane thickness along healing periods. The thickness of membranes in both control and experimental groups were seen to decrease with time and no significant difference (*p* > 0.05) was found between the two groups along the observation periods.

The data of membrane thickness measured during healing periods showed that the thickness of both control and experimental group decreased with time (Figure 5). Statistical analysis revealed that there was no significant difference (*p* > 0.05) between the two groups in all observation periods.

## 4. Discussion

### 4.1. In Vitro Anticellularity Evaluation

This study explored the potential of bovine cortical bone collagen to be used as a xenogeneic membrane material in humans so that it was important that the material be nonimmunogenic. One of the main causes of human immune response was the cellular components in xenogeneic material. Therefore, in order to use animal-derived tissues, decellularization was the first and most important issue [9]. The result of in vitro anticellularity evaluation showed that there was no residual cell (osteoblasts and osteocytes) seen in the histology section of DFDBCBM. This result confirmed that manufacturing process of DFDBCBM had removed retained cells in bovine cortical bone which made the DFDBCBM, to some extent, nonantigenic.

### 4.2. Cytotoxicity Test

The result of MTT Assay showing statistically lower optical density of fibroblast exposed to DFDBCBM-conditioned medium than those to normal medium at all time point might indicate that DFDBCB membrane, to a certain extent, cytotoxic. However, the increment in percentage of viable fibroblast along the three time points, all of which were above 60%, suggested that there was no cell death but it is likely that DFDBCBM-conditioned medium might have caused some inhibition in cell growth. These findings might be associated with the activity of hydrogen peroxide or HCL or both still retained within DFDBCBM after wash-out procedure. The low porosity nature of cortical bone may need more cleaning time to completely wash out the chemical agents absorbed during manufacturing process.

### 4.3. In Vivo Immunogenic Response Evaluation

Following the implantation of biomaterials in vivo, host reactions incorporated a combination of many processes including blood-material interactions, provisional matrix formation, inflammation (acute then chronic), development of granulation tissue, foreign body reaction, and fibrous capsule development [10, 11]. The provisional matrix was rich in cytokines, growth factors, and chemoattractants that are capable of recruiting cells of the innate immune system to the injury site. The degree of these responses was dependent on the extent of injury during the implantation procedure. The presence of neutrophils (PMNs) characterized the acute inflammatory response [12].

The higher PMN infiltration in DFDBCBM group compared to BPCM group observed during the first week of healing confirmed that the material had evoked inflammatory response. These findings could be attributable to two possible factors. First, it could be associated with residual components of processing agent for DFDBCB membrane. Second, the DFDBCBM might be slightly contaminated which may be caused by improper handling of the package of the membrane during implantation procedure or possibly associated with the sterilization procedure during manufacturing process. However, the downward pattern of PMN cells infiltration in DFDBCBM and BPCM group might indicate that inflammatory response decreased with time in both groups which was important for tissue integration.

The result of macrophage and lymphocytes counting which was higher in DFDBCBM group in early postimplantation periods again showed that the material had evoked inflammatory response. However, no statistical differences were found between the two groups further down the healing phase. This suggested that both membranes did not cause either excessive or prolonged immune response. Biocompatible implanted materials usually demonstrated early resolution of chronic inflammatory response being no longer than two weeks and being confined to implantation site [12]. This was important for the membranes to be able to have tissue integration and hence avoid early membrane degradation in later period.

### 4.4. Tissue Response Evaluation

Normal tissue response to implantation of biomaterial followed physiologic process of healing which consisted of cellular infiltration, release of chemokines from cells (1–5 days), recruitment of tissue repair cells (5–15 days), and fibrous encapsulation and granulation tissue formation (3-4 weeks) [12]. After the resolution of acute and chronic inflammatory responses had occurred, granulation tissue was seen and confirmed by the presence of macrophages, fibroblast infiltration, and neovascularization in the new tissue. Granulation tissue may be a precursor to fibrous capsule formation and is separated from the implanted biomaterial device by the cellular components of the foreign body reaction (consisting of macrophages and foreign body giant cells or FBGCs).

Based on this, we examined stages of tissue response using grading scale of capsule quantity and quality around membranes. The four stages of tissue response were initial phase or early tissue repair (7 days), intermediate phase or proliferative stage (14 and 21 days), and late phase or maturation of fibrous capsule (28 days). The higher scores in initial and intermediate phase indicated the lag in healing process, while in late phase the higher scores indicated the speedy maturation of the fibrous capsule.

The result of evaluation of capsule quantity showed that the number of fibroblasts in DFDBCBM group was relatively lower than the control group which indicated lag in early tissue repair. This phenomenon might be caused by slightly extended inflammatory response in DFDBCBM group in the immune response evaluation above in which inflammatory cells infiltration in DFFDBCBM group was relatively higher than BPCM group at the end of day 7 after implantation although it was not statistically significant.

The result of evaluation of capsule quality showed that both BPCM and DFDBCBM groups exhibited constant increase in capsule quality without any significance difference between the two groups along the observation period. This indicated that normal fibrous encapsulation, along with normal capsule maturation, had occurred in both groups without any signs of prolonged inflammations.

The response of tissues to a foreign material was much the same as the standard response to tissue injury; however, inflammation and macrophage activation did not resolve at the later stages and persistence of inflammatory cells, in particular macrophages, occurred [13]. Macrophages had been shown to respond and naturally bound to almost all biomaterials once implanted, including collagen [14]. It had been demonstrated that macrophages participate in the degradation of biomaterials by the release of a variety of enzymes [15], mediators of degradation such as reactive oxygen intermediates (ROIs), enzymes, and acid between the cell membrane and biomaterial surface [16]. Macrophages could fuse and became foreign body giant cells (FBGCs), which were observed at biomaterial-tissue interface of implanted devices and tissue engineering scaffolds [17]. It was suggested that implant sites that had a greater number of macrophages and foreign body giant cells had more fibrosis and encapsulation of the biomaterials [18]. This phenomenon was evident in the result of this study in which macrophages were seen to populate the surface of the membranes, inside porosities of BPCM structures and at DFDBCBM cleavage areas which confirmed the role of macrophages in membrane biodegradation. The results also showed that no statistical difference was found regarding the interface tissue quality in both types of membrane, which logically meant that there was no excessive number of macrophages and FBGC in the interface tissue of DFDBCBM. This could be attributable to the facts that both types of membrane were composed of bovine fibrillar collagen type-I although they were taken from different parts of the body.

### 4.5. Biodegradation Evaluation

The biodegradation evaluation in this study used measurement of membrane thickness as parameter of membrane integrity after subcutaneous implantation. The result of the evaluation showed that membrane thickness decreased gradually with time in both groups and without any statistical difference between them until the end of observation period. This result confirmed that DFDBCBM had a biodegradation rate comparable with that of BPCM, at least until 28 days after implantation.

The result of biodegradation evaluation also showed that there was a difference in the behavior or pattern of degradation between the two types of membrane. The BPCM degradation was observed to occur at the periphery of the structure demonstrated by concavities at the membrane surface. On the contrary, DFDBCBM degradation was observed to be characterized by formation of cleavage of the membrane leading to membrane fragmentation. This could be the manifestation of mixed tissue response occurring after implantation of biologic scaffold characterized by incorporation through the graft openings, combined with encapsulation around the remaining material [19]. From the result of biodegradation evaluation it was also shown that until 28 days of observation period both membranes still retained more than halves their initial dimensions. In order to play its role as a barrier, absorbable membranes should remain for at least three to four weeks [20]. It was supported by result of a study which showed that spontaneous healing and closure of the wound were completed within 3-4 weeks after resorption process of exposed absorbable membranes [21].

## 5. Conclusion

Based on the result of this study it was concluded that demineralized freeze-dried bovine cortical bone membrane or DFDBCBM has some potential for application as guided bone regeneration membrane, as it elicited normal tissue response and underwent gradual biodegradation when implanted subcutaneously. However, some level of in vitro toxicity and increased immunogenic responses in early phase of healing postsubcutaneous implantation showed that further study is required to optimize its biocompatibility to fulfill all requirements for GBR membrane.

## Figures and Tables

**Table 1:** Grading scale for tissue response analysis of the membrane [7].

Reaction zone	Response	Score
Capsule quantitatively	Thickness rating:	
	(i) l–4 fibroblasts	4
	(ii) 5–9 fibroblasts	3
	(iii) 10–30 fibroblasts	2
	(iv) >30 fibroblasts	1
	(v) Not applicable	0

Capsule qualitatively	(i) Capsule tissue is fibrous, mature, not dense, resembling connective or fat tissue in the noninjured regions	4
	(ii) Capsule tissue is fibrous but immature, showing fibroblasts and little collagen	3
	(iii) Capsule tissue is granulous and dense, containing both fibroblasts and many inflammatory cells	2
	(iv) Capsule consists of masses of inflammatory cells with little or no signs of connective tissue organization	1
	(v) Cannot be evaluated because of infection or other factors not necessarily related to the material	0

Interface qualitatively	(i) Fibroblasts contact the implant surface without the presence of macrophages or foreign body giant cells	4
	(ii) Scattered foci of macrophages and foreign body cells are present	3
	(iii) One layer of macrophages and foreign body cells is present	2
	(iv) Multiple layers of macrophages and foreign body cells are present	1
	(v) Cannot be evaluated because of infection or other factors not necessarily related to the material	0
